# *Plasmodium falciparum* gametocyte dynamics after pyronaridine–artesunate or artemether–lumefantrine treatment

**DOI:** 10.1186/s12936-018-2373-7

**Published:** 2018-06-04

**Authors:** Johanna M. Roth, Patrick Sawa, George Omweri, Victor Osoti, Nicodemus Makio, John Bradley, Teun Bousema, Henk D. F. H. Schallig, Pètra F. Mens

**Affiliations:** 1 Department of Medical Microbiology, Laboratory for Clinical Parasitology, Academic Medical Center, Meibergdreef 9, 1105 AZ Amsterdam, The Netherlands; 2 Human Health Division, International Centre of Insect Physiology and Ecology, Mbita Point, Kenya; 3 Medical Research Council Tropical Epidemiology Group, London School of Hygiene and Tropical Medicine, London, United Kingdom; 4 Radboud Institute for Health Sciences, Radboud University Medical Center, Nijmegen, The Netherlands

**Keywords:** *Plasmodium falciparum*, Gametocytes, Artemether–lumefantrine, Pyronaridine–artesunate

## Abstract

**Background:**

Artemisinin-based combinations differ in their impact on gametocyte prevalence and density. This study assessed female and male gametocyte dynamics after treating children with uncomplicated *Plasmodium falciparum* malaria with either pyronaridine–artesunate (PA) or artemether–lumefantrine (AL).

**Methods:**

Kenyan children with uncomplicated *Plasmodium falciparum* malaria were included and randomly assigned to PA or AL treatment. Filter paper blood samples were collected as a source of RNA for quantitative reverse-transcription PCR (qRT-PCR) and nucleic acid sequence based amplification (QT-NASBA) to detect female gametocytes (targeting *Pfs25* mRNA). Male gametocytes were detected by qRT-PCR (targeting *PfMGET* mRNA). Duration of gametocyte carriage, the female and male gametocyte response and the agreement between qRT-PCR and QT-NASBA were determined.

**Results:**

The mean duration of female gametocyte carriage was significantly longer for PA (4.9 days) than for AL (3.8 days) as estimated by QT-NASBA (P = 0.036), but this difference was less clear when determined by *Pfs25* qRT-PCR (4.5 days for PA and 3.7 for AL, P = 0.166). qRT-PCR based female gametocyte prevalence decreased from 100% (75/75) at baseline to 6.06% (4/66) at day 14 in the AL group and from 97.7% (83/85) to 13.9% (11/79) in the PA group. Male gametocyte prevalence decreased from 41.3% (31/75) at baseline to 19.7% (13/66) at day 14 in the AL group and from 35.3% (30/85) to 22.8% (18/79) in the PA group. There was good agreement between *Pfs25* qRT-PCR and QT-NASBA female gametocyte prevalence (0.85, 95% CI 0.82–0.87).

**Conclusions:**

This study indicates that female gametocyte clearance may be slightly faster after AL compared to PA. Male gametocytes showed similar post-treatment clearance between study arms. Future studies should further address potential differences between the post-treatment transmission potential after PA compared to AL.

*Trial registration* This study is registered at clinicaltrials.gov under NCT02411994. Registration date: 8 April 2015. https://clinicaltrials.gov/ct2/show/NCT02411994?term=pyronaridine-artesunate&cond=Malaria&cntry=KE&rank=1

**Electronic supplementary material:**

The online version of this article (10.1186/s12936-018-2373-7) contains supplementary material, which is available to authorized users.

## Background

Since artemisinin-based combination therapy (ACT) became widely adopted as first-line treatment for uncomplicated *Plasmodium falciparum* malaria, it considerably contributed to the decline of the disease burden [1–4]. However, resistance against commonly used artemisinin-based combinations is rising in South-East Asia and the potential spread to African countries is a major public health concern [5, 6]. New drugs are under development that offer possible alternatives to currently used artemisinin-based combinations. One of these alternatives is the fixed-dose combination therapy pyronaridine–artesunate (PA), which is found to be well tolerated and efficacious for the treatment of uncomplicated *P. falciparum* malaria and the blood stage of *Plasmodium vivax* malaria [7–12]. Mild and transient increases in transaminases are the main safety concern [11].

So far, the effect of PA on the transmission stages of *P. falciparum* (gametocytes), has not been extensively studied in the clinical setting. In vitro data are contradicting: a strong gametocytocidal effect of pyronaridine against stage II–IV gametocytes has been found [13], but was not confirmed elsewhere [14]. Delves et al. reported activity of pyronaridine against stage V gametocytes in vitro, although only at concentrations close to cytotoxic levels, suggesting limited clinical relevance [15, 16]. With the increasing efforts to reduce malaria transmission, it becomes highly important to evaluate not only the potential of ACT to cure the asexual stage of the parasite, but also their effect on gametocytes. ACT is generally effective against asexual stages and immature gametocytes, but its activity against mature gametocytes is limited [14, 17–19]. However, differences between artemisinin-based combinations in the gametocyte response after treatment exist. A recent meta-analysis showed that the appearance of gametocytaemia in patients without gametocytes at baseline was lower after artemether–lumefantrine (AL) and artesunate-mefloquine (AS-MQ) compared to dihydroartemisinin–piperaquine (DP) and artesunate-amodiaquine (AS-AQ) [20]. Among patients with gametocytes at baseline, clearance was faster after AS-MQ and slower after DP, compared to AL. This meta-analysis by the Worldwide Antimalarial Resistance Network (WWARN) hypothesized that the non-artemisinin partner drug is a relevant determinant for differences in the post-treatment gametocyte response.

To accurately evaluate the gametocyte response after ACT treatment, molecular tools are informative since post-treatment gametocyte densities are often below the detection threshold of microscopy [21]. Quantitative Nucleic Acid Sequence Based Amplification (QT-NASBA) is a sensitive and reliable technique for the detection of submicroscopic gametocytes, targeting the female-specific *Pfs25* [22, 23]. Recently, a sex-specific quantitative reverse transcriptase PCR (qRT-PCR) has been developed and evaluated, differentiating between female (*Pfs25*) and male (*PfMGET*) gametocytes [24]. This differentiation may be important, because the minority male population (normally 3–5 females to 1 male) was shown in vitro to be more sensitive than females to a range of anti-malarial drugs [15]. Thus, faster clearance of the male gametocyte population during or after treatment might sterilize the infection, while the female-dominated gametocyte density may not be reduced to the same extent [25]. The sex-specific qRT-PCR can be used to investigate both male and female gametocyte dynamics in clinical trials.

In this study, the QT-NASBA and qRT-PCR based female specific gametocyte response after PA-treatment was compared to that after AL. Furthermore, qRT-PCR was used to evaluate and compare male and female gametocyte dynamics. Finally, the agreement between *Pfs25* qRT-PCR and QT-NASBA for the detection of female gametocytes was determined.

## Methods

### Study design

This observational study was part of a phase III randomized clinical trial investigating the efficacy and safety of PA compared to AL in Kenyan children with uncomplicated *P. falciparum* malaria [26]. The study was conducted at St. Jude’s Clinic, Mbita, Western Kenya, from October 2015 to June 2016 and from January to August 2017. Ethical approval was obtained from the Ethical Review Committee of the Kenya Medical Research Institute (KEMRI) (NON-SSC no. 479, registered at clinicaltrials.gov under NCT02411994). Children aged 6 months to 12 years seeking care at the clinic were eligible to participate if they were living within 10 km range from the study clinic and had microscopically confirmed *P. falciparum* mono-infection with a parasitaemia between 1000 and 200,000/µl. Exclusion criteria were signs and symptoms of complicated malaria, non-*P. falciparum* or mixed *Plasmodium* infection, a history of hepatic and/or renal impairment, a haemoglobin (Hb) concentration < 6 g/dL, severe malnutrition (defined as having a weight-for-age or height-for-age z-score of < − 3) [27], having received anti-malarial therapy in the previous 2 weeks, known hypersensitivity to artemisinins, previous participation in this study, current participation in other anti-malarial drug intervention studies or not being available for follow-up. Written informed consent from a parent or guardian was required for study participation, assent was sought from children able to understand the study.

### Procedures

Study participants were randomized to receive a 3-day course of either artemether–lumefantrine (AL, Novartis, Basel, Switzerland) or pyronaridine–artesunate (PA, Shin Poong Pharmaceutical Company, Seoul, South Korea). All study staff except the pharmacists responsible for drug administration were blinded to treatment allocation. Dosing was body-weight dependent and drugs were administered according to manufacturer’s instructions (Additional file 1) with food (mandazi—a type of fried bread) or milk. For PA, children < 20 kg received granules dissolved in lemonade. Children ≥ 20 kg received the tablet formulation. In the AL group, all children received tablets.

Participants returned to the study clinic on 1, 2, 3, 7, 14, 28 and 42 days after start of treatment. Blood samples were taken by finger-prick at all time-points. Hb was determined on day 0, 3, 7 and 28 by HemoCue (Ängelholm, Sweden). Giemsa-stained thick smears were used for determination and counting of asexual parasites and gametocytes, according to WHO procedures [28]. Thick-and-thin blood smears were prepared and read by local expert microscopists. A slide was considered negative when 100 high-power fields were examined at 1000 × magnification and no parasites were observed. Parasitaemia was determined from thick smears by counting the number of parasites against 200 leukocytes, with the assumption of 8000 leukocytes/µl blood. When the number of parasites after counting 200 leukocytes was < 100, counting continued up to 500 leukocytes.

Female specific *Pfs25* QT-NASBA and sex-specific *Pfs25* and *PfMGET* qRT-PCR were used for gametocyte detection on day 0, 3, 7 and 14. To perform these assays, 2 × 50 µl finger-prick blood was collected on Whatman 903 protein saver cards (GE Healthcare, Chicago, USA), dried at room temperature for 24 h, packed individually with silica and stored at − 20 °C until shipment to the Netherlands. Nucleic acid extraction was done by Nuclisens EasyMag (bioMérieux, Marcy-l’Étoile, France) and DNA/RNA was stored at − 70 °C. QT-NASBA was performed as previously described [22], with minor modifications. The reaction mixture (5 µl) and sample (2.5 µl) were incubated for 2 min at 65 °C and 2 min at 41 °C. Enzyme was added (2.5 µl) and the reaction was allowed to run for 30 min at 41 °C. Quantification was done using standard curves of 10^3^ to 10^−1^ gametocytes/µl, which were produced from in vitro cultures as reported [22]. qRT-PCRs were performed as previously described, using sex-specific standard curves (10^3^ to 10^−2^ gametocytes/µl) for quantification [24]. To produce these separate standard curves, male and female gametocytes were isolated by fluorescence activated cell sorting using a transgenic parasite line expressing male and female specific fluorescence markers [29]. Samples were declared negative for both QT-NASBA and qRT-PCR if the estimated gametocytaemia was < 0.02 gametocytes/µl (1 gametocyte/50 µl sample) [30].

### Outcomes

The primary outcome was the mean duration of female gametocyte carriage in the PA arm compared to the AL arm, based on QT-NASBA. Secondary outcomes were: the qRT-PCR based mean duration of female gametocyte carriage, the QT-NASBA and qRT-PCR based female gametocyte circulation time, the QT-NASBA based area under the curve (AUC) of female gametocyte density over time (gametocytes/µl^−1^ days), and gametocyte prevalence and density on day 3, 7 and 14 as determined by QT-NASBA and *Pfs25*/*PfMGET* qRT-PCR. Finally, the agreement between QT-NASBA and *Pfs25* qRT-PCR for the detection but not quantification of female gametocytes was determined.

### Statistical analysis

Stata software version 14.0 (Stata Corporation, Texas, USA) and SAS version 9.4 (SAS Institute Inc, NC, USA) were used for statistical analyses. A deterministic compartmental model, as previously published, was fitted to determine the duration of female gametocyte carriage and gametocyte circulation times [31]. The AUC was determined as described previously and log10-transformed [32]. Linear regression was used to compare the log AUC in the PA group to that in the AL group, adjusting for log10-transformed baseline gametocyte density. The Wilcoxon rank-sum test was used for between-group comparisons of gametocyte density on day 0, 3, 7 and 14. A Chi square or Fisher’s exact test was used to compare between-group gametocyte prevalences on day 0, 3, 7 and 14. The agreement between *Pfs25* qRT-PCR and QT-NASBA was determined by calculating the weighted concordance correlation coefficient (CCC) based on variance components, taking repeated measures into account [33, 34].

## Results

### Study population and baseline characteristics

**Fig. 1:**
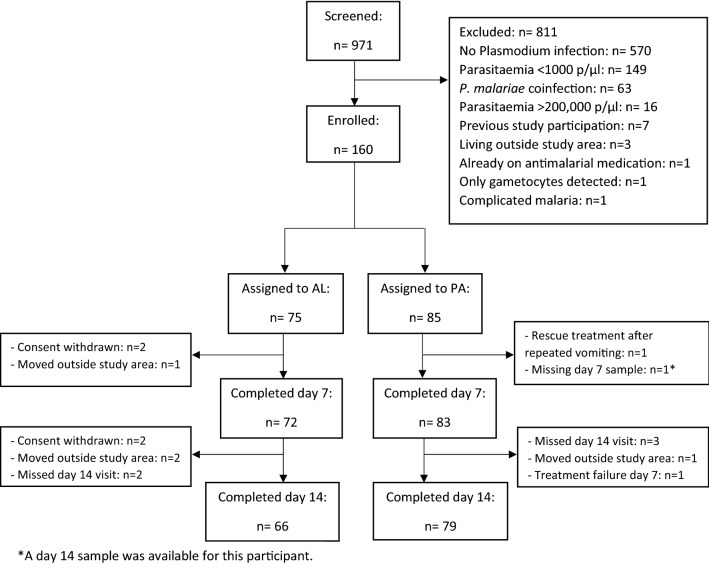
Participant flow. Schematic presentation of patient screening, inclusion and follow-up for the present study

A consecutive subset of 160 children from the main clinical trial participated in the present study. Of these 160 participants, 85 received PA and 75 received AL (Fig. 1). Nine participants did not complete follow-up (day 14) in the AL group: four withdrew consent, three moved away from the study area and two missed their day 14 visit. In the PA group, six participants did not complete follow-up: three missed their day 14 visit, one discontinued due to repeated vomiting, one moved away from the study area and one had a treatment failure on day 7.

Baseline characteristics were similar between intervention groups (Table 1). QT-NASBA and *Pfs25* qRT-PCR based female gametocyte prevalence and density at baseline were comparable and higher than *PfMGET* qRT-PCR male estimates. As expected, microscopy based prevalence was lower compared to the molecular methods and children with microscopy confirmed gametocytes at baseline had significantly higher *Pfs25* QT-NASBA gametocyte density compared to those without microscopically detected gametocytes (P < 0.001, Wilcoxon rank-sum test).

**Table 1:** Baseline characteristics of study participants at enrollment

	Pyronaridine–artesunate	Artemether–lumefantrine
N	85	75
Male^a^	54.1 (46/85)	49.3 (37/75)
Age (years)^b^	7.0 (4.2–9.0)	6.0 (3.4–9.8)
Hb (g/dL)^c^	11.8 (11.4–12.2)	11.7 (11.2–12.2)
Temperature (°C)^c^	37.6 (37.4–37.9)	37.4 (37.1–37.7)
Fever (temperature > 37.5 °C)^a^	55.3 (47/85)	48.0 (36/75)
Asexual parasite density (p/µl)^b^	28,800 (12,000–68,640)	29,280 (12,000–67,680)
Gametocyte prevalence—microscopy^a^	2.35 (2/85)	5.33 (4/75)
Gametocyte prevalence—QT-NASBA^a^	95.3 (81/85)	94.7 (71/75)
Gametocyte density—QT-NASBA (p/µl)^b^	3.23 (0.68–18.1)	6.36 (1.19–22.2)
Gametocyte prevalence—*Pfs25* qRT-PCR^a^	97.7 (83/85)	100 (75/75)
Gametocyte density—*Pfs25* qRT-PCR (p/µl)^b^	2.88 (0.85–5.24)	1.94 (0.77–5.35)
Gametocyte prevalence—*PfMGET* qRT-PCR^a^	35.3 (30/85)	41.3 (31/75)
Gametocyte density—*PfMGET* qRT-PCR (p/µl)^b^	0.94 (0.11–14.1)	0.48 (0.21–10.1)

Data are: ^a^ Percentage % (n/N), ^b^ Median and IQR or ^c^ Mean and 95% CI

### Gametocyte carriage after PA and AL

**Fig. 2:**
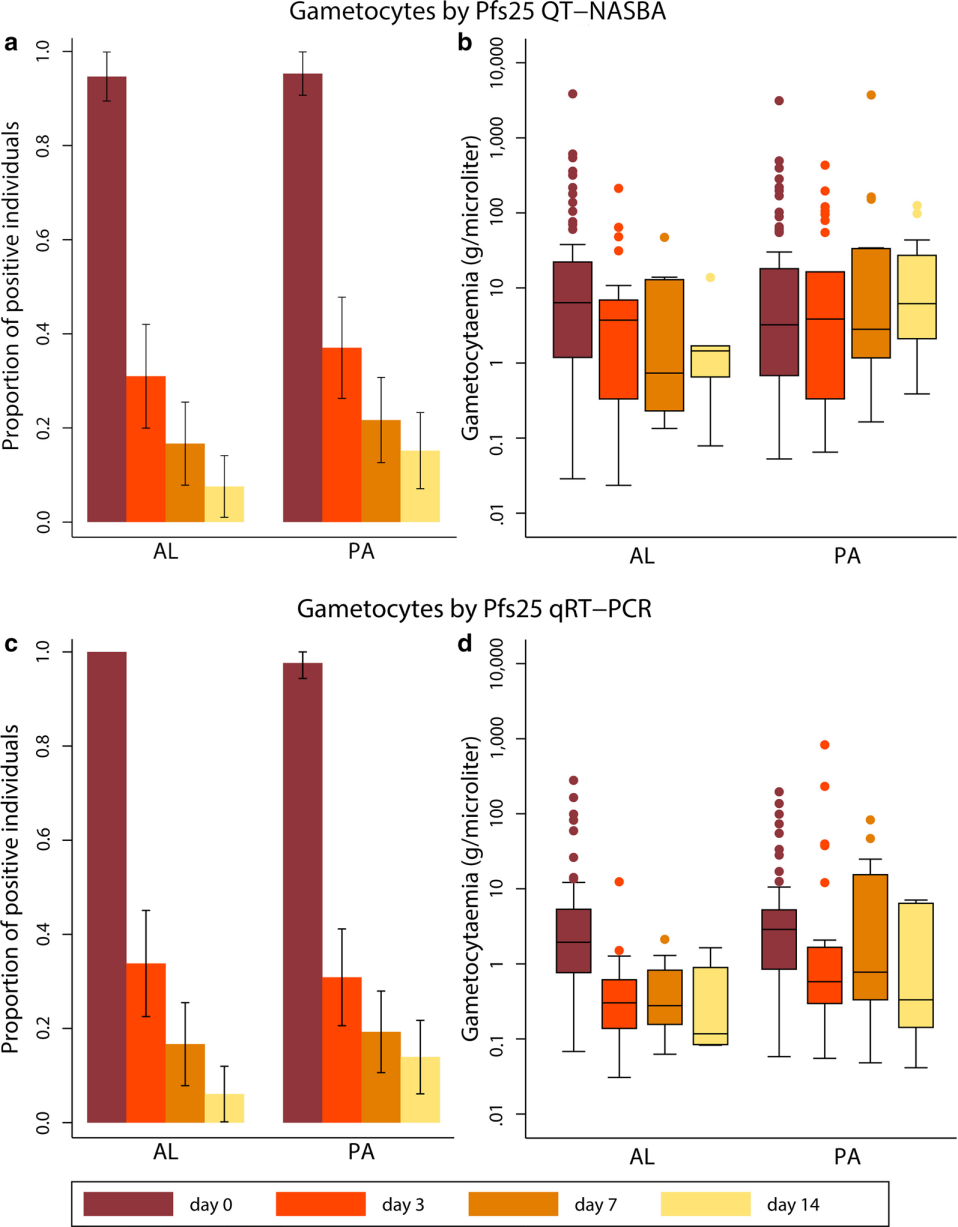
Female gametocytes by *Pfs25* QT-NASBA and qRT-PCR. **a** Gametocyte prevalence determined by QT-NASBA. **b** Gametocyte density determined by QT-NASBA. **c** Gametocyte prevalence determined by qRT-PCR. **d** Gametocyte density determined by qRT-PCR. 95% confidence intervals are presented for prevalences. Density is presented as median (IQR) for gametocyte-positive individuals only. Samples were considered negative if gametocyte levels were < 0.02/µl. *AL* artemether–lumefantrine, *PA* pyronaridine–artesunate

QT-NASBA based female gametocyte prevalence at day 3 was 37.0% (30/81) in the PA group and 31.0% (22/71) in the AL group (P = 0.433). At day 7, prevalence decreased to 21.7% (18/83) in the PA group and 16.7% (12/72) in the AL group (P = 0.430). Prevalence at day 14 was 15.2% (12/79) in the PA group and 7.58% (5/66) in the AL group (P = 0.156). Female gametocyte prevalence estimates were highly similar when assessed by *Pfs25* qRT-PCR (Fig. 2, Table 2).

**Table 2:** Female and male gametocyte prevalence and density

	Pyronaridine–artesunate	Artemether–lumefantrine	*P* value*
*Pfs25* QT-NASBA prevalence, % (no./No.)			
Day 0	95.3 (81/85)	94.7 (71/75)	1.000
Day 3	37.0 (30/81)	31.0 (22/71)	0.433
Day 7	21.7 (18/83)	16.7 (12/72)	0.430
Day 14	15.2 (12/79)	7.58 (5/66)	0.156
*Pfs25* QT-NASBA density, median (IQR)^a^			
Day 0	3.23 (0.68–18.1)	6.36 (1.19–22.2)	0.229
Day 3	3.86 (0.33–16.4)	3.73 (0.33–6.91)	0.630
Day 7	2.82 (1.17–33.5)	0.74 (0.23–12.9)	0.150
Day 14	6.19 (2.10–27.2)	1.45 (0.65–1.69)	0.092
*Pfs25* qRT-PCR prevalence, % (no./No.)			
Day 0	97.7 (83/85)	100 (75/75)	0.499
Day 3	30.9 (25/81)	33.8 (24/71)	0.699
Day 7	19.3 (16/83)	16.7 (12/72)	0.674
Day 14	13.9 (11/79)	6.06 (4/66)	0.122
*Pfs25* qRT-PCR density, median (IQR)^a^			
Day 0	2.88 (0.85–5.24)	1.94 (0.77–5.35)	0.568
Day 3	0.58 (0.30–1.66)	0.30 (0.14–0.62)	0.039
Day 7	0.78 (0.33–15.5)	0.28 (0.16–0.83)	0.126
Day 14	0.33 (0.14–6.42)	0.12 (0.08–0.90)	0.322
*PfMGET* qRT-PCR prevalence (%) (no./No.)			
Day 0	35.3 (30/85)	41.3 (31/75)	0.433
Day 3	34.6 (28/81)	36.6 (26/71)	0.792
Day 7	31.3 (26/83)	30.6 (22/72)	0.918
Day 14	22.8 (18/79)	19.7 (13/66)	0.652
*PfMGET* qRT-PCR density, median (IQR)^b^			
Day 0	0.94 (0.11–14.1)	0.48 (0.21–10.1)	0.920
Day 3	2.55 (0.21–11.0)	0.82 (0.17–5.07)	0.341
Day 7	3.23 (0.21–8.95)	1.22 (0.19–11.5)	0.551
Day 14	1.81 (0.30–11.2)	1.51 (0.66–6.91)	0.779
Total qRT-PCR prevalence, % (no./No.)			
Day 0	97.7 (83/85)	100 (75/75)	0.499
Day 3	42.0 (34/81)	43.7 (31/71)	0.841
Day 7	32.5 (27/83)	31.9 (23/72)	0.920
Day 14	25.3 (20/79)	21.2 (14/66)	0.560
Total qRT-PCR density, median (IQR)^c^			
Day 0	3.23 (0.86–7.54)	2.24 (0.91–6.13)	0.642
Day 3	1.20 (0.24–11.0)	1.06 (0.11–5.21)	0.248
Day 7	3.47 (0.26–15.5)	1.69 (0.27–12.3)	0.631
Day 14	1.66 (0.30–14.6)	1.34 (0.55–7.00)	0.662

* P-values represent between group differences. Prevalence differences were tested with the Chi squared or Fisher’s Exact test. Differences in gametocyte density were tested with the Wilcoxon rank-sum test

Data included: ^a^ female gametocyte positive individuals, ^b^ male gametocyte positive individuals, ^c^ male and/or female gametocyte positive individuals

While prevalence estimates were comparable between QT-NASBA and qRT-PCR, some differences in density measurements were observed. As can be seen in Fig. 2, the decrease in density over time is clearer by qRT-PCR as compared to QT-NASBA, especially in the PA group. In the AL group, the QT-NASBA based female gametocyte density decreased from a median of 6.36 gametocytes/µl (IQR 1.19–22.2) at baseline to 1.45 gametocytes/µl (IQR 0.65–1.69) at day 14. However, in the PA group, the median female gametocyte density estimated by QT-NASBA in gametocyte positive samples did not decrease over time and even appeared to increase slightly: 3.23 gametocytes/µl (IQR 0.68–18.1) at baseline versus 6.19 gametocytes/µl (IQR 2.11–27.2) at day 14. Given that the median gametocyte density is only determined over gametocyte positive individuals, the median density on day 3, 7 and 14 is not necessarily assessed over the same individuals used to determine the baseline gametocyte density. To investigate whether this apparent increase over time in the PA group was due to an absolute increase within individuals, the median QT-NASBA based gametocyte density on day 0 for individuals still positive on day 14 was calculated and found to be 113.0 (IQR 21.0–252). Thus, the median female gametocyte density for participants in the PA group gametocyte positive on day 14 decreased from baseline to day 14 and the apparent rise in density as estimated by QT-NASBA could not be explained by an absolute increase within individuals, but is rather a difference between the population positives on day 0 and that on day 14.

**Fig. 3:**
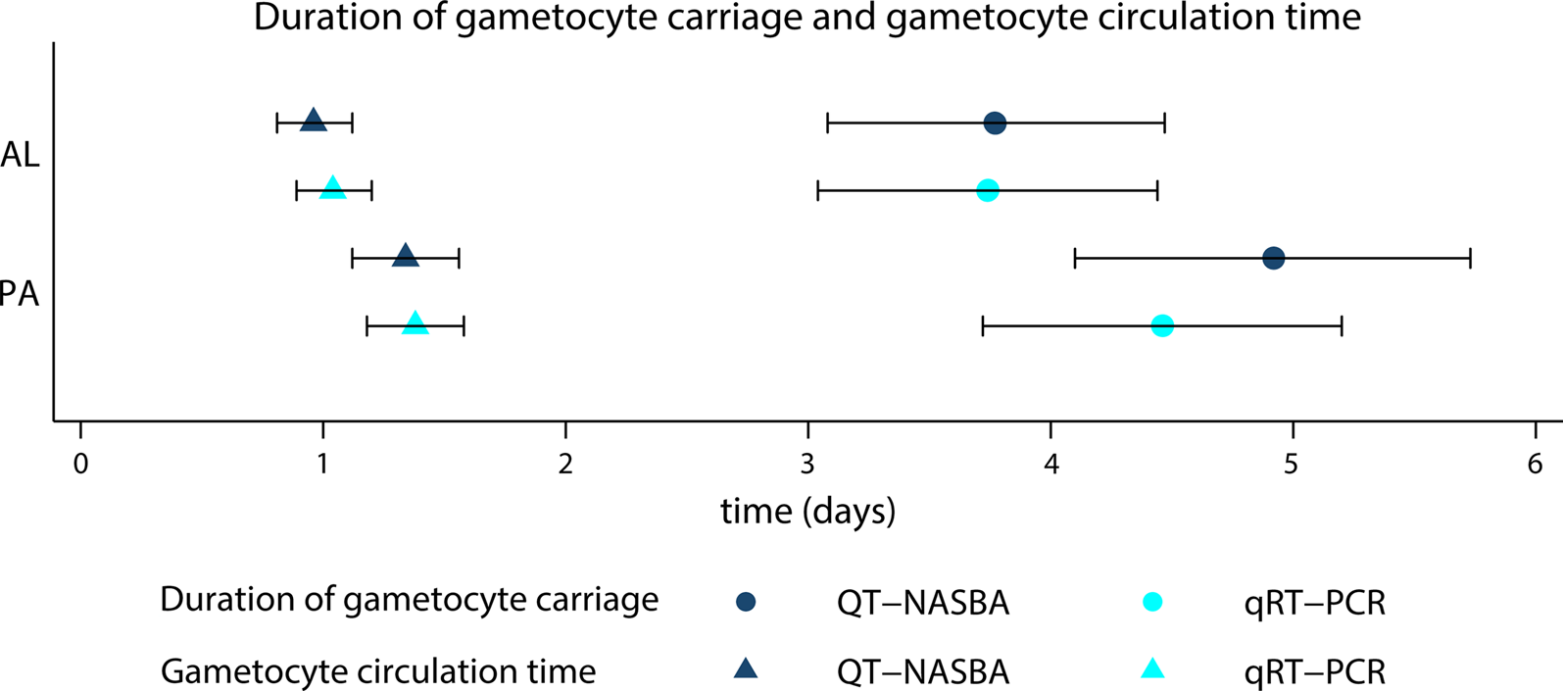
Duration of female gametocyte carriage and gametocyte circulation time. Rounds represent the mean duration of female gametocyte carriage (in days) and their error bars the upper and lower limit of the 95% CI. Triangles represent the mean female gametocyte circulation time (in days) and their error bars the upper and lower limit of the 95% CI. *AL* artemether–lumefantrine, *PA* pyronaridine–artesunate

The mean duration of female gametocyte carriage as estimated by QT-NASBA was significantly longer in the PA group (4.92 days, 95% CI 4.10–5.73), compared to the AL group (3.77 days, 95% CI 3.08–4.47) (P = 0.036). By qRT-PCR, the mean duration of female gametocyte carriage was also longer in the PA group (4.46 days, 95% CI 3.72–5.20), compared to the AL group (3.74 days, 95% CI 3.04–4.44), but this difference was not significant (P = 0.166). Similarly, the QT-NASBA based mean gametocyte circulation time was longer for PA (1.34 days, 95% CI 1.12–1.56) compared to AL (0.96 days, 95% CI 0.81–1.12) (P = 0.003). This was also the case for the qRT-PCR based mean gametocyte circulation time (PA: 1.38 days, 95% CI 1.18–1.58 and AL: 1.04 days, 95% CI 0.89–1.20, P = 0.004) (Fig. 3 and Table 3). The AUC, on the other hand, was not different between treatment arms (Table 3, P = 0.617 after adjustment for baseline gametocyte density), possibly explained by the slower gametocyte clearance in the PA group, but considerable contribution of the higher baseline gametocyte density to the AUC in the AL group.

**Table 3:** Estimates female gametocyte carriage, circulation time and area under the curve (AUC)

	Pyronaridine–artesunate	Artemether–lumefantrine	P-value
*Pfs25* QT-NASBA			
Duration of gametocyte carriage (days), mean (95% CI)	4.92 (4.10–5.73)	3.77 (3.08–4.47)	0.036
Circulation time (days), mean (95% CI)	1.34 (1.12–1.56)	0.96 (0.81–1.12)	0.003
AUC (g/µl^−1^ day), median (IQR)	0.52 (0.105–2.771)	0.79 (0.177–3.150)	0.617*
*Pfs25* qRT-PCR			
Duration of gametocyte carriage (days), mean (95% CI)	4.46 (3.72–5.20)	3.74 (3.04–4.44)	0.166
Circulation time (days), mean (95% CI)	1.38 (1.18–1.58)	1.04 (0.89–1.20)	0.004

* Adjusted for baseline gametocyte density

### Effect of PA and AL on male and female gametocytes

**Fig. 4:**
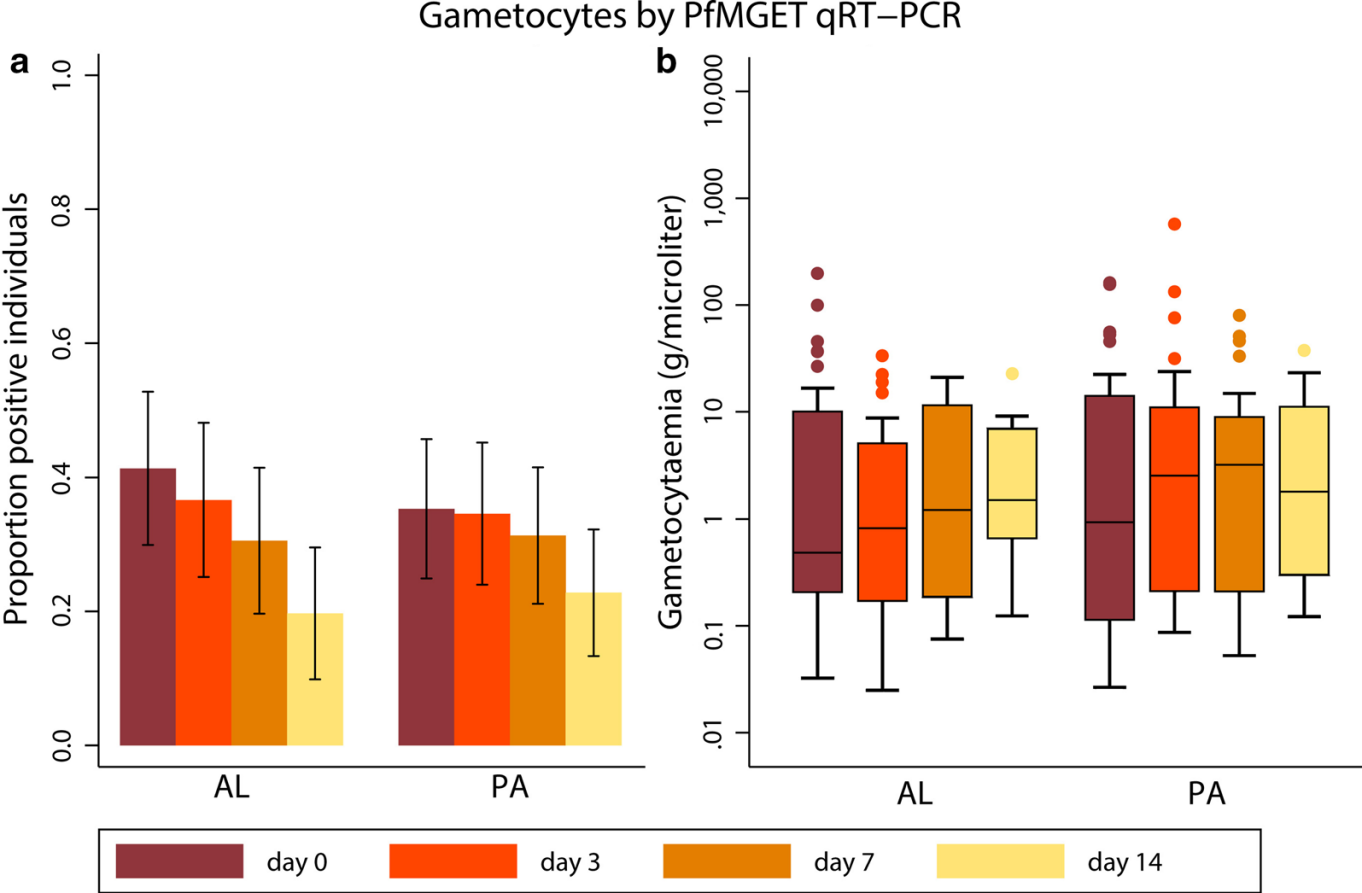
Male gametocytes by *PfMGET* qRT-PCR. **a** Gametocyte prevalence, including 95% confidence intervals. **b** Gametocyte density, presented as median (IQR) for gametocyte-positive individuals only. Samples were considered negative if gametocyte levels were < 0.02/µl. *AL* artemether–lumefantrine, *PA* pyronaridine–artesunate

qRT-PCR was used to differentiate between male and female gametocyte responses after PA and AL. At baseline, female gametocytes were detected in 97.7% (83/85) of participants in the PA group and 100% (75/75) of the participants in the AL group (P = 0.499). This prevalence decreased to 13.9% (11/79) on day 14 in the PA group and 6.06% (4/66) in the AL group (P = 0.122) (Fig. 2). At baseline, the male prevalence was lower compared to the female prevalence: 35.3% (30/85) in the PA group and 41.3% (31/75) in the AL group (P = 0.433). In both groups the decrease in male gametocyte prevalence was less substantial than that of female prevalence (male gametocyte prevalence 22.8% (18/79) in the PA group and 19.7% (13/66) in the AL group on day 14 (P = 0.652) (Fig. 4).

The median female density decreased from 2.88 (IQR 0.85–5.24) gametocytes/µl at baseline to 0.58 (IQR 0.30–1.66) on day 3 in the PA group. In the AL group, female baseline density was 1.94 gametocytes/µl (IQR 0.77–5.35), which decreased to 0.30 (IQR 0.14–0.62) on day 3. By day 14, median female gametocyte density was 0.33 (IQR 0.14–6.42) in the PA group and 0.12 (IQR 0.08–0.90) in the AL group (P = 0.322) (Table 2). Median male density, on the other hand, did not decrease over time and even appears to increase slightly. Similar to the analysis of QT-NASBA density in the PA group as described above, the apparent increase of male gametocytes over time was investigated. The median gametocyte density on day 0 for individuals still positive on day 14 was calculated and found to be 9.44 (IQR 0.18–45.7) in the PA group and 10.1 (IQR 0.81–36.5) in the AL group. Thus, the median gametocyte density for participants gametocyte positive on day 14 decreased from baseline to day 14 in both treatment groups (P = 0.01 for both PA and AL groups, Wilcoxon signed rank test). This illustrates also for male gametocytes that the apparent rise in density could not be explained by an absolute increase within individuals, but is rather a difference between the population positives on day 0 and that on day 14.

### Agreement between *Pfs25* qRT-PCR and QT-NASBA

The agreement between binary female prevalence outcomes of *Pfs25* qRT-PCR and QT-NASBA was calculated using the CCC variance components method. Only subjects with a complete dataset were included (140 participants and 560 samples). Out of 560, 12 samples were positive by QT-NASBA but not by qRT-PCR. Similarly, 10 samples were positive by qRT-PCR but negative by QT-NASBA. The two tests were in agreement for the remaining 538 samples (209 positive and 329 negative). The CCC was 0.85 (95% CI 0.82–0.87), indicating good agreement between *Pfs25* qRT-PCR and QT-NASBA for the detection of female gametocytes.

## Discussion

This is the first paper describing kinetics of submicroscopic gametocytes after PA treatment using molecular detection methods in comparison with the most widely used first-line treatment for malaria in Africa, AL. The duration of female gametocyte carriage and gametocyte circulation time appeared to be slightly longer for PA compared to AL. There were no indications that PA or AL preferentially cleared male gametocytes.

The failure of conventional anti-malarials, including ACT, to clear circulating mature gametocytes may allow persisting malaria transmission in the week(s) following treatment [35]. Gametocyte clearance time may thus be a relevant indicator of the transmission-blocking potential of anti-malarial drugs. Although it has been observed that some persisting gametocytes may not be viable [36], current evidence suggests that a comparison of anti-malarial drugs on gametocytocidal properties would reach similar conclusions on their relative transmission blocking effects [18, 35–37]. The microscopy-based gametocyte clearance time has previously been compared between PA and AL, but no difference between the two drugs was found [7, 10]. Since microscopy is notoriously insensitive for gametocyte detection, molecular methods provide more accurate estimates of post treatment gametocytaemia [38]. Importantly, it has been shown that submicroscopic gametocytes may allow onward transmission to mosquitoes [39]. In the present study, gametocytes were detected by microscopy in only 3.75% (6/160) of study participants at baseline, compared to 95.0% (152/160) by QT-NASBA. This contrast is even higher than in previous studies and emphasizes the underestimation of gametocyte prevalence by microscopy [38].

Different effects of ACT on the gametocyte response have previously been reported and in a recent meta-analysis AL was shown to be better in preventing the microscopic occurrence of gametocytes shortly after treatment compared to DP or AS-AQ [20]. A point of caution when interpreting the results of this meta-analysis, is the fact that sensitivities of parasites to the drugs fluctuated over the years and drug efficacy is setting dependent. However, there is agreement in literature that, based on both microscopy as well as molecular gametocyte detection, the duration of gametocyte carriage is significantly shorter after AL treatment, compared to DP [20, 35]. In the present study, the duration of gametocyte carriage and gametocyte circulation time were surprisingly short compared to other studies in the same area [35, 37]. While the day 3 QT-NASBA female gametocyte prevalence was 31.0% (22/71) for AL and 37.0% (30/81) for PA, others reported day 3 QT-NASBA prevalences > 50% after AL treatment among those positive at baseline [35, 37]. Previous studies in sub-Saharan Africa, using the same model to assess gametocyte clearance and circulation time, found a duration of gametocyte carriage after AL of 12.4 days in a trial with similar inclusion criteria to the present study [40] and of 19.7 days in a trial including patent gametocyte carriers [41]. These gametocyte carriage estimates are 3–5 fold longer compared to the present study. A possible explanation for this observation is the relatively low median gametocyte density at baseline in the present study. Alternatively, the process of storing and extracting RNA from filter papers may have resulted in a suboptimal yield and underestimated gametocyte prevalence during follow up. Despite the shorter clearance estimates compared to other reports, there are no indications that this observation affected the comparison between PA and AL in the present study.

Baseline prevalence of female gametocytes (estimated by qRT-PCR) was 98.8% (158/160), while male baseline prevalence was only 38.1% (61/160). This is in contrast to the data presented by Stone et al. [24], from the same study site, where both female and male prevalence were 100% as estimated by the same qRT-PCR. However, the study by Stone et al. included only participants with microscopically detectable gametocytes, while being gametocyte positive by microscopy was uncommon in the present study. This resulted in median baseline qRT-PCR-based gametocyte densities of 2.9/µl (PA) and 1.9/µl (AL) for female gametocytes and 0.9/µl (PA) and 0.5/µl (AL) for males. Working with such low densities, with presumably a female biased sex-ratio at baseline, it is not unlikely that part of the samples with low density female gametocytaemia at baseline had male densities below the detection threshold, which may explain the difference in baseline prevalence between male and female gametocytes.

No evidence of faster male compared to female gametocyte clearance was found. In fact, the present data suggest that even though the proportion of participants with male gametocytes at baseline was lower than that with female gametocytes, males may actually be cleared slower. Previous studies that examined gametocyte sex ratio after DP or SP-AQ alone or with primaquine observed that during the course of follow-up gametocyte sex ratios became more female-biased while primaquine initially resulted in a male-biased sex ratio [24, 36]. In microscopy-based studies a female biased gametocyte response after various artemisinin-based combinations was commonly observed [42, 43]. In vitro results also indicate a more pronounced effect of most anti-malarial drugs on male compared to female gametocytes. For example, the percentage inhibition of activation by artemether and artesunate was found to be approximately 39 and 10 times higher, respectively, for males than for females [15]. The difference between the qRT-PCR used in the present study and the in vitro system used by Delves et al. is that the latter evaluates the gametocytes’ ability to form gametes rather than the presence of mRNA. Whether the qRT-PCR can detect mRNA from nonviable gametocytes is unknown [40]. Both the in vitro and the mRNA results can be accurate if male gametocytes are more affected by the ACT than females, but remain present in the circulation during the time of sampling as intact nonviable gametocytes [24]. Thus, despite the clear added value of molecular techniques like QT-NASBA and qRT-PCR, functional assays that determine gametocyte fitness or infectivity remain crucial in assessing transmission-blocking properties of anti-malarial drugs.

The apparent increase in male density (and female density in the PA arm as estimated by QT-NASBA) could not be explained by an absolute increase within individuals, but rather reflects a difference between the population positives on day 0 and that on day 14. Stone et al. performed a similar analysis and reported a small decrease of male density after DP treatment (from 3.8/µl at baseline to 0.9/µl at day 7) [24]. Both studies had low baseline male gametocyte density, but estimates were approximately five times higher in the study by Stone et al. Baseline densities close to the detection limit could lead to an increase in density by chance and this could possibly explain the difference in density over time between the two studies. Additionally, a study by Dicko et al. found a higher baseline density of male gametocytes and showed a more distinct decrease over time compared to both the present study and Stone et al. [24, 36].

A good level of agreement between QT-NASBA and *Pfs25* qRT-PCR female gametocyte prevalence was observed. This confirms data from a previous study where both assays were shown to be suitable to detect and quantify submicroscopic levels of gametocytes, although the reproducibility of qRT-PCR was found to be better than that of QT-NASBA [30].

A limitation of the present study is that gametocyte infectiousness to mosquitoes could not be established. This was due to an infection of the established mosquito colony with *Microsporidia* species, which has been shown to inhibit the survival of *Plasmodium* in mosquitoes [44]. Since only mosquito feeding assays can provide evidence on the transmissibility of gametocytes, an assessment of infectivity could not be done. Future studies should further address potential differences between the post-treatment transmission potential after PA compared to AL.

## Conclusions

This study provides important data on the submicroscopic gametocyte response after PA compared to AL treatment of uncomplicated *P. falciparum* malaria. These data may contribute to estimates of impact differences between artemisinin-based combinations, for example based on a model that demonstrated a higher reduction of clinical episodes using long-acting combinations in high transmission settings, while combinations with shorter half-lifes but more pronounced gametocytocidal effects were shown to be more suitable for low-transmission settings [45].

## Additional file

**Additional file 1.** Weight-based dosing of pyronaridine–artesunate and artemether–lumefantrine.

## References

[1] Bhatt S, Weiss DJ, Cameron E, Bisanzio D, Mappin B, Dalrymple U (2015). The effect of malaria control on *Plasmodium falciparum* in Africa between 2000 and 2015. Nature.

[2] Maude RJ, Pontavornpinyo W, Saralamba S, Aguas R, Yeung S, Dondorp AM (2009). The last man standing is the most resistant: eliminating artemisinin-resistant malaria in Cambodia. Malar J..

[3] Maude RJ, Socheat D, Nguon C, Saroth P, Dara P, Li G (2012). Optimising strategies for *Plasmodium falciparum* malaria elimination in Cambodia: primaquine, mass drug administration and artemisinin resistance. PLoS ONE.

[4] Nosten F, van Vugt M, Price R, Luxemburger C, Thway K, Brockman A (2000). Effects of artesunate-mefloquine combination on incidence of *Plasmodium falciparum* malaria and mefloquine resistance in western Thailand: a prospective study. Lancet.

[5] Ashley EA, Dhorda M, Fairhurst RM, Amaratunga C, Lim P, Suon S (2014). Spread of artemisinin resistance in *Plasmodium falciparum* malaria. NEJM..

[6] Fairhurst RM, Dondorp AM. Artemisinin-resistant *Plasmodium falciparum* malaria. Microbiol Spectr. 2016;4:El10-0013-2016. 10.1128/microbiolspec.EI10-0013-2016 PMC4992992 27337450

[7] Tshefu AK, Gaye O, Kayentao K, Thompson R, Bhatt KM, Sesay SSS (2010). Efficacy and safety of a fixed-dose oral combination of pyronaridine–artesunate compared with artemether–lumefantrine in children and adults with uncomplicated *Plasmodium falciparum* malaria: a randomised non-inferiority trial. Lancet.

[8] Poravuth Y, Socheat D, Rueangweerayut R, Uthaisin C, Pyae Phyo A, Valecha N (2011). Pyronaridine–artesunate versus chloroquine in patients with acute *Plasmodium vivax* malaria: a randomized, double-blind, non-inferiority trial. PLoS ONE.

[9] Rueangweerayut R, Phyo AP, Uthaisin C, Poravuth Y, Binh TQ, Ph D (2012). Pyronaridine–artesunate versus mefloquine plus artesunate for malaria. NEJM..

[10] Kayentao K, Doumbo OK, Pénali LK, Offianan AT, Bhatt KM, Kimani J (2012). Pyronaridine–artesunate granules versus artemether–lumefantrine crushed tablets in children with *Plasmodium falciparum* malaria: a randomized controlled trial. Malar J..

[11] Duparc S, Borghini-fuhrer I, Craft JC, Arbe-barnes S, Miller RM, Shin C (2013). Safety and efficacy of pyronaridine–artesunate in uncomplicated acute malaria: an integrated analysis of individual patient data from six randomized clinical trials. Malar J..

[12] Sagara I, Beavogui AH, Zongo I, Soulama I, Borghini-fuhrer I, Fofana B (2016). Safety and efficacy of re-treatments with pyronaridine–artesunate in African patients with malaria: a substudy of the WANECAM randomised trial. Lancet Infect Dis..

[13] Chavalitshewinkoon-Petmitr P, Pongvilairat G, Auparakkitanon S, Wilairat P (2000). Gametocytocidal activity of pyronaridine and DNA topoisomerase II inhibitors against multidrug-resistant *Plasmodium falciparum* in vitro. Parasitol Int.

[14] Adjalley SH, Johnston GL, Li T, Eastman RT, Ekland EH, Eappen AG (2011). Quantitative assessment of *Plasmodium falciparum* sexual development reveals potent transmission-blocking activity by methylene blue. Proc Natl Acad Sci USA.

[15] Delves MJ, Ruecker A, Straschil U, Lelièvre J, Marques S, López-Barragán MJ (2013). Male and female *Plasmodium falciparum* mature gametocytes show different responses to antimalarial drugs. Antimicrob Agents Chemother.

[16] Lelièvre J, Almela MJ, Lozano S, Miguel C, Franco V, Leroy D (2012). Activity of clinically relevant antimalarial drugs on *Plasmodium falciparum* mature gametocytes in an ATP bioluminescence “transmission blocking” assay. PLoS ONE.

[17] Kumar N, Zheng H (1990). Stage-specific gametocytocidal effect in vitro of the antimalaria drug qinghaosu on *Plasmodium falciparum*. Parasitol Res.

[18] Targett G, Drakeley C, Jawara M, von Seidlein L, Coleman R, Deen J (2001). Artesunate reduces but does not prevent posttreatment transmission of *Plasmodium falciparum* to *Anopheles gambiae*. J Infect Dis.

[19] White NJ (2008). The role of anti-malarial drugs in eliminating malaria. Malar J..

[20] WWARN Gametocyte Study Group (2016). Gametocyte carriage in uncomplicated *Plasmodium falciparum* malaria following treatment with artemisinin combination therapy: a systematic review and meta-analysis of individual patient data. BMC Med..

[21] Schneider P, Bousema T, Omar S, Gouagna L, Sawa P, Schallig H (2006). (Sub)microscopic *Plasmodium falciparum* gametocytaemia in Kenyan children after treatment with sulphadoxine-pyrimethamine monotherapy or in combination with artesunate. Int J Parasitol.

[22] Schneider P, Schoone G, Schallig H, Verhage D, Telgt D, Eling W (2004). Quantification of *Plasmodium falciparum* gametocytes in differential stages of development by quantitative nucleic acid sequence-based amplification. Mol Biochem Parasitol.

[23] Mens PF, Sawa P, Van Amsterdam SM, Versteeg I, Omar SA, Schallig HDFH (2008). A randomized trial to monitor the efficacy and effectiveness by QT-NASBA of artemether–lumefantrine versus dihydroartemisinin-piperaquine for treatment and transmission control of uncomplicated *Plasmodium falciparum* malaria in western Kenya. Malar J..

[24] Stone W, Sawa P, Lanke K, Rijpma S, Oriango R, Nyaurah M (2017). A molecular assay to quantify male and female *Plasmodium falciparum* gametocytes: results from 2 randomized controlled trials using primaquine for gametocyte clearance. J Infect Dis.

[25] White N, Ashley E, Recht J, Delves M, Ruecker A, Smithuis F (2014). Assessment of therapeutic responses to gametocytocidal drugs in *Plasmodium falciparum* malaria. Malar J..

[26] Roth JM, Sawa P, Makio N, Omweri G, Osoti V, Okach S (2018). Pyronaridine–artesunate and artemether–lumefantrine for the treatment of uncomplicated *Plasmodium falciparum* malaria in Kenyan children: a randomized controlled non-inferiority trial. Malar J..

[27] WHO. Child growth standards and the identification of severe acute malnutrition in infants and children. Geneva: World Health Organization; 2009. http://www.who.int/nutrition/publications/severemalnutrition/9789241598163/en/. Accessed 14 Dec 2017. 24809116

[28] WHO. Basic malaria microscopy—part I: Learner’s guide. 2 ed. Geneva: World Health Organization; 2010. http://www.who.int/malaria/publications/atoz/9241547820/en/. Accessed 12 Jan 2018.

[29] Lasonder E, Rijpma SR, Van Schaijk BCL, Hoeijmakers WAM, Kensche PR, Gresnigt MS (2016). Integrated transcriptomic and proteomic analyses of *P. falciparum* gametocytes: molecular insight into sex-specific processes and translational repression. Nucleic Acids Res.

[30] Pett H, Gonçalves BP, Dicko A, Nébié I, Tiono AB, Lanke K (2016). Comparison of molecular quantification of *Plasmodium falciparum* gametocytes by Pfs25 qRT-PCR and QT-NASBA in relation to mosquito infectivity. Malar J..

[31] Bousema T, Okell L, Shekalaghe S, Griffin JT, Omar S, Sawa P (2010). Revisiting the circulation time of *Plasmodium falciparum* gametocytes: molecular detection methods to estimate the duration of gametocyte carriage and the effect of gametocytocidal drugs. Malar J..

[32] Méndez F, Muñoz Á, Plowe C (2006). Use of area under the curve to characterize transmission potential after antimalarial treatment. Am J Trop Med Hyg.

[33] Carrasco JL, King TS, Chinchilli VM (2009). The concordance correlation coefficient for repeated measures estimated by variance components. J Biopharm Stat.

[34] Pan Y, Rose CE, Haber M, Ma Y, Carrasco JL, Stewart B (2013). Assessing agreement of repeated binary measurements with an application to the CDC’s anthrax vaccine clinical trial. Int J Biostat..

[35] Sawa P, Shekalaghe SA, Drakeley CJ, Sutherland CJ, Mweresa CK, Baidjoe AY (2013). Malaria transmission after artemether–lumefantrine and dihydroartemisinin-piperaquine: a randomized trial. J Infect Dis.

[36] Dicko A, Roh ME, Diawara H, Mahamar A, Soumare HM, Lanke K (2018). Efficacy and safety of primaquine and methylene blue for prevention of *Plasmodium falciparum* transmission in Mali: a phase 2, single-blind, randomised controlled trial. Lancet Infect Dis..

[37] Bousema JT, Schneider P, Gouagna LC, Drakeley CJ, Tostmann A, Houben R (2006). Moderate effect of artemisinin-based combination therapy on transmission of *Plasmodium falciparum*. J Infect Dis.

[38] Bousema T, Drakeley C (2011). Epidemiology and infectivity of *Plasmodium falciparum* and *Plasmodium vivax* gametocytes in relation to malaria control and elimination. Clin Microbiol Rev.

[39] Schneider P, Bousema JT, Gouagna LC, Otieno S, Van De Vegte-Bolmer M, Omar SA (2007). Submicroscopic *Plasmodium falciparum* gametocyte densities frequently result in mosquito infection. Am J Trop Med Hyg.

[40] Eziefula AC, Bousema T, Yeung S, Kamya M, Owaraganise A, Gabagaya G (2014). Single dose primaquine for clearance of *Plasmodium falciparum* gametocytes in children with uncomplicated malaria in Uganda: a randomised, controlled, double-blind, dose-ranging trial. Lancet Infect Dis..

[41] Gonçalves BP, Tiono AB, Ouédraogo A, Guelbéogo WM, Bradley J, Nebie I (2016). Single low dose primaquine to reduce gametocyte carriage and *Plasmodium falciparum* transmission after artemether–lumefantrine in children with asymptomatic infection: a randomised, double-blind, placebo-controlled trial. BMC Med..

[42] Sowunmi A, Balogun ST, Gbotosho GO, Happi CT (2009). *Plasmodium falciparum* gametocyte sex ratios in symptomatic children treated with antimalarial drugs. Acta Trop.

[43] Gbotosho GO, Sowunmi A, Okuboyejo TM, Happi CT, Michael OS, Folarin OA (2011). *Plasmodium falciparum* gametocyte carriage, emergence, clearance and population sex ratios in anaemic and non-anaemic malarious children. Mem Inst Oswaldo Cruz.

[44] Koella JC, Lorenz L, Bargielowski I (2009). Microsporidians as evolution-proof agents of malaria control?. Adv Parasitol.

[45] Okell LC, Cairns M, Griffin JT, Ferguson NM, Tarning J, Jagoe G (2014). Contrasting benefits of different artemisinin combination therapies as first-line malaria treatments using model-based cost-effectiveness analysis. Nat Commun..

