# Supplementary material for: Plasmodium falciparum gametocyte dynamics after pyronaridine–artesunate or artemether–lumefantrine treatment
Source: Malar J. 2018 Jun 4;17:223. doi: 10.1186/s12936-018-2373-7 (PMC5987563; doi:10.1186/s12936-018-2373-7)
Supplement: Supplementary file 1 — Additional file 1. Weight-based dosing of pyronaridine–artesunate and artemether–lumefantrine. [file 12936_2018_2373_MOESM1_ESM.pdf]

Additional file 1: Weight-based dosing of treatments

Total duration of treatment for both pyronaridine-artesunate and artemether-lumefantrine: 3 days.

**Pyronaridine-Artesunate**	
**Patient weight**	**Number of sachets per day**
≥5 to <8 kg	1 (total course: 3 sachets)
8 to <15 kg	2 (total course: 6 sachets)
15 to <20 kg	3 (total course: 9 sachets)
**Patient weight**	**Number of tablets per day**
20 to <24 kg	1 (total course: 3 tablets)
24 to <45 kg	2 (total course: 6 tablets)

**Artemether-Lumefantrine**	
**Patient weight**	**Number of tablets**
≥5 to <15 kg	1 tablet as single initial dose, followed by 1 tablet after 8 hours, and then 1 tablet twice a day (morning and evening) for the following 2 days (total course: 6 tablets)
15 to <25 kg	2 tablets as single initial dose, followed by 2 tablets after 8 hours, and then 2 tablets twice a day (morning and evening) for the following 2 days (total course: 12 tablets)
25 to <35 kg	3 tablets as single initial dose, followed by 3 tablets after 8 hours, and then 3 tablets twice a day (morning and evening) for the following 2 days (total course: 18 tablets)
≥35 kg	4 tablets as single initial dose, followed by 4 tablets after 8 hours, and then 4 tablets twice a day (morning and evening) for the following 2 days (total course: 24 tablets)
